# Dermatomyositis: focus on cutaneous features, etiopathogenetic mechanisms and their implications for treatment

**DOI:** 10.1007/s00281-025-01054-9

**Published:** 2025-08-06

**Authors:** Hammad Ali, Aretha On, Enze Xing, Catherine Shen, Victoria P. Werth

**Affiliations:** 1 Corporal Michael J. Crescenz Veterans Affairs Medical Center, Philadelphia, PA USA; 2 Department of Dermatology, Perelman School of Medicine, University of Pennsylvania, Philadelphia, PA USA

**Keywords:** Dermatomyositis, Amyopathic dermatomyositis, Pathogenetic mechanisms for dermatomyositis, Etiology of dermatomyositis, Cutaneous manifestations of dermatomyositis, Treatments for dermatomyositis

## Abstract

Dermatomyositis (DM) is an infrequently encountered idiopathic inflammatory myopathy distinguished by distinctive cutaneous manifestations and/or progressive muscle weakness. This review provides an updated exploration of DM, emphasizing cutaneous features, etiopathogenesis, and therapeutic implications. DM presents a heterogeneous spectrum, ranging from classic forms involving both skin and muscle to clinically amyopathic DM, which lacks significant muscle involvement but carries risks like interstitial lung disease (ILD) and malignancy. Recent advances in understanding DM pathogenesis underscore the roles of myositis-specific autoantibodies, type I interferons, and cytokine dysregulation in disease activity and clinical outcomes. Specific antibodies such as anti-Mi-2, anti-TIF1γ, and anti-MDA5 define subtypes of DM, aiding diagnosis, prognosis, and tailored management strategies. While conventional immunosuppressive therapies like glucocorticoids and antimalarials form the cornerstone of treatment, many cases remain refractory, particularly involving chronic skin disease. Emerging targeted therapies, including Janus kinase inhibitors and monoclonal antibodies, show promise in addressing type I interferon-driven pathways and refractory symptoms. Future research aims to refine diagnostic criteria, integrate biomarkers, utilize more robust outcome measures, and develop targeted therapeutics to improve outcomes while minimizing treatment-related toxicity. This review consolidates current knowledge and highlights the need for a multidisciplinary, individualized approach to managing DM, focusing on both established and novel treatment avenues.

## Introduction

Dermatomyositis (DM) is an infrequently encountered, idiopathic inflammatory myopathy (IIM) noted for the presence of distinctive cutaneous manifestations and/or progressive muscle weakness [1, 2]. As a member of the IIMs, which include polymyositis (PM), antisynthetase syndrome (ASyS), and inclusion body myositis (IBM), DM uniquely combines features of inflammatory muscle disease with specific skin findings. However, DM presents with a broad clinical spectrum, ranging from classic presentations with both skin and muscle involvement to clinically amyopathic dermatomyositis (CADM), to severe systemic involvement, including interstitial lung disease (ILD) and an elevated risk of malignancy [3]. This variability makes DM a complex and challenging disease to diagnose and manage.

Although the underlying mechanisms of DM are not yet fully elucidated, current evidence suggests that genetic susceptibility, environmental triggers, and immune dysregulation all contribute to its development. Myositis-specific autoantibodies (MSAs) appear to play a key role in delineating clinical subtypes and may potentially be involved in the pathophysiology of the disease as well. [4]. Emerging evidence supports the utility of MSAs in diagnostic assessment, while also offering prognostic insights and informing individualized therapeutic approaches [4, 5]. These autoantibodies correlate with specific clinical phenotypes and are linked to complications such as heightened malignancy risk, ILD, and severe cutaneous involvement [4, 5]. The link between DM and malignancy, especially in adults, underscores the importance of early detection and comprehensive evaluation in these patients [6].

The diagnosis of dermatomyositis can be challenging due to its diverse clinical presentations and the overlap of its signs and symptoms with other connective tissue diseases [7]. Furthermore, DM can present without significant muscle weakness, known as CADM, complicating the clinical picture. However, advancements in serologic testing and imaging techniques have enhanced our ability to diagnose DM more accurately.

Despite its rarity, DM is linked to considerable morbidity and mortality, primarily driven by its associated complications. The risk of malignancy in adult patients, the potential for ILD and rapidly progressive ILD (RP-ILD), and the chronicity of skin disease in many cases make DM a condition that requires a comprehensive and multidisciplinary approach to management [8, 9]. Treatment strategies primarily involve immunosuppressive and immunomodulatory therapies, but responses are variable. Some patients experience a chronic disease course marked by cycles of remission and relapse, with skin symptoms often remaining stubbornly resistant to treatment—even when muscle inflammation is well controlled [10, 11]. This heterogeneity highlights the necessity for enhanced insight into the underlying pathophysiological mechanisms of DM and the advancement of more precise, targeted treatment strategies.

This article provides an in-depth examination of dermatomyositis, with a particular emphasis on the pathogenesis of its cutaneous manifestations, associated clinical features, diagnostic challenges, and the evolving spectrum of therapeutic strategies. By consolidating current evidence and highlighting areas of ongoing research, this review seeks to offer a comprehensive resource for clinicians and researchers alike, fostering a better understanding of this complex and multifaceted disease.

### Clinical features

From Bohan and Peter’s initial classification back in 1975, requiring muscle involvement for the diagnosis of DM, to the revised criteria proposed by Sontheimer in 2002 for the inclusion of CADM (amyopathic and hypomyopathic) we have come a long way in our understanding of the disease [1–3]. The EULAR/ACR now allows identification of amyopathic DM with the myositis criteria [12]. Ongoing efforts are further refining the skin criteria for DM [13]. It is still unclear whether classic and amyopathic diseases are a spectrum of the same disease process or represent totally different disease processes with completely different pathogenesis. The skin findings are identical for both [14].

DM can be classified into several subtypes based on clinical features, associated conditions, and disease onset. This classification helps in understanding the disease's heterogeneity and guiding management and prognosis.

DM classification can be done on the basis of clinical features [1–4, 12]:

1. **Classic DM**
  **Description**: Classic DM is characterized by the presence of both characteristic skin findings and muscle inflammation.
  **Features**:
    Heliotrope rash (violaceous rash around the eyes)
    Gottron's papules (erythematous papules over knuckles)
    Gottron’s sign (flat erythema over bony prominences, including knuckles but also extending to other areas like elbows and knees.)
    Symmetrical proximal muscle weakness (e.g., challenges with transitioning from sitting to standing or ascending stairs)
    Elevated muscle enzymes (creatine kinase, aldolase)
    Abnormal electromyography (EMG), muscle MRI and muscle biopsy findings

2. **Clinically amyopathic dermatomyositis (CADM)**
  **Description**: CADM, also known as dermatomyositis sine myositis, presents with hallmark cutaneous features of DM in the absence of clinically apparent muscle involvement.. When lacking both clinical and laboratory evidence of muscle disease it is subclassified as amyopathic and if clinical symptoms are absent however there are laboratory abnormalities, then it is subclassified as hypomyopathic variant.
  **Features**:
    All classic skin features of DM (e.g., heliotrope rash, Gottron's papules/sign)
    No muscle weakness
    Muscle biopsy, EMG, and muscle enzymes may be mildly abnormal
  **Significance**: CADM patients may still have systemic manifestations like ILD. The disease can progress to classic DM, so monitoring is essential, especially early in the disease. Usually, patients can present initially with skin manifestations with later development of muscle inflammation, so the diagnosis of CADM is only made after 6 months of cutaneous disease in the absence of muscle involvement. One prospective database demonstrated that only 5% of CADM patients progressed to classic DM and the median time to conversion was 6.3 years (9).

3. **Juvenile Dermatomyositis (JDM)**: Given the scope of this article the juvenile variant will not be covered in detail.
  **Description**: JDM presents in children and adolescents and shares many features with adult DM but often includes additional systemic involvement.
  **Features**:
    Similar cutaneous findings as in adult DM
    Calcinosis (deposition of calcium in the skin and muscles) is more common
    Gastrointestinal vasculopathy, leading to abdominal pain and GI bleeding
    Greater risk of lipodystrophy
  **Significance**: Generally, a better prognosis than adult DM, but with a risk of significant morbidity due to calcinosis and other systemic complications.

4. **Dermatomyositis associated with malignancy**
  **Description**: A subset of adult DM patients have an underlying malignancy, often diagnosed around the same time as DM or shortly thereafter.
  **Features** [15]:
    Classic cutaneous and muscular features of DM.
    Older age and male gender.
    Higher incidence of certain cancers; Lung cancer is the most prevalent type of cancer in men with DM; Breast and gynecological cancers are the most prevalent types of cancer in women with DM; Gastrointestinal malignancies are common in both sexes.
    Presence of anti-TIF1γ (anti-p155/140) and anti-NXP2 antibodies are implicated in increased malignancy risk.

5. **Overlap syndromes**
  **Description**: In overlap syndromes, DM coexists with other connective tissue diseases, including systemic lupus erythematosus (SLE), systemic sclerosis (SSc), or rheumatoid arthritis (RA).
  **Features**:
    Cutaneous features of DM
    Additional symptoms from overlapping autoimmune diseases
    Presence of specific antibodies (e.g., anti-U1-RNP in mixed connective tissue disease)

Another way of classifying DM involves identifying specific autoantibodies, however given that a substantial number of patient’s can be seronegative the absence of antibodies does not preclude the diagnosis of DM. Culprit antibodies and associated clinical features will be reviewed here [4, 10, 16]:

#### Myositis-specific antibodies (MSAs)

Different subtypes of DM are associated with specific MSAs. For example, the association of anti-Mi-2 with classic DM, whilst anti-MDA5 is often linked to clinically amyopathic DM and is associated with ILD.

ILD is a common complication in DM, especially in those with specific antibodies (e.g., anti-synthetase antibodies or anti-MDA5), and can significantly affect prognosis.

Classification by autoantibody/clinical phenotype can help tailor clinical management strategies, guiding both prognostic assessments and therapeutic approaches.

- **Anti-Mi-2**: Targets a nuclear DNA helicase involved in transcription.
  o **Clinical Features**: Associated with"classic"DM presentations, including heliotrope rash, Gottron's papules/sign, shawl sign, and periungual erythema. Patients typically have raised creatine kinase (CK) levels and proximal, symmetric muscle involvement. Anti-Mi-2 is generally associated with a favorable treatment and prognosis.
- **Anti-TIF1-γ (same as anti-p155/140 on Western Blot)**: Targets a tumor suppressor protein acting as a transcriptional corepressor.
  o **Clinical Features**: Significantly correlated with an elevated risk of malignancy in adults. This antibody is linked to severe, photosensitive cutaneous manifestations, such as psoriasiform plaques, palmar hyperkeratosis, and atrophic hypopigmented patches with telangiectasias. Characteristic “red on white” poikiloderma and ovoid palatal patch can be seen in seropositive patients [17]. It is also associated with hypomyopathic disease and esophageal dysmotility.
- **Anti-MDA5 (formerly anti-CADM140)**: Targets an RNA-specific helicase implicated in mediating antiviral immune responses.
  o **Clinical Features**: Associated with CADM and a greater risk of developing ILD, particularly RP-ILD. Commonly presents with unique cutaneous findings, including ulcerative skin lesions, painful palmar papules (inverse Gottron papules), and panniculitis. There is less association with malignancy, but patients with anti-MDA5 antibodies often exhibit a severe, refractory disease course with significant pulmonary involvement.
- **Anti-NXP2 (formerly anti-MJ)**: Targets a nuclear matrix protein involved in transcription regulation.
  o **Clinical Features**: Associated with severe myositis affecting both proximal and distal muscles, peripheral edema, and a higher prevalence of calcinosis cutis, particularly in children. This autoantibody is linked to a higher risk of malignancy in adults, though no such association has been observed in pediatric cases. This subtype may also involve gastrointestinal vasculopathy leading to bleeding.
- **Anti-SAE1/2**: Recognizes a small ubiquitin-like modifier–activating enzyme that functions in the posttranslational modification of proteins.
  o **Clinical Features**: Associated with severe skin disease, subtle initial muscle involvement, but progressive muscle involvement over time, often with dysphagia. Characteristic “angel wing” rash on the back can be seen in these patients. Patients can exhibit systemic manifestations such as fever and unintended weight loss. Some data suggests a potential link with mild ILD in certain populations. The association with malignancy is still unclear.
- **Antisynthetase antibodies:** These are a subset of MSAs that target aminoacyl-tRNA synthetases, which are enzymes involved in protein synthesis [18]. These antibodies are strongly associated with a condition known as antisynthetase syndrome (ASyS), a subtype of IIMs that is characterized by a constellation of clinical features: myositis, ILD, arthritis, Raynaud's phenomenon, fever, and"mechanic's hands"[18]. However, mechanics hands are frequently seen in DM as well [19].

- There are several types of antisynthetase antibodies, each targeting a different aminoacyl-tRNA synthetase. The most common and well-studied antisynthetase antibody is anti-Jo-1. Others include anti-PL-7 (Threonyl-tRNA Synthetase), anti-PL-12 (Alanyl-tRNA Synthetase), anti-EJ (Glycyl-tRNA Synthetase), anti-OJ (Isoleucyl-tRNA Synthetase), anti-KS (Asparaginyl-tRNA Synthetase), anti-Ha (Histidyl-tRNA Synthetase, another form of Anti-Jo-1), and anti-Zo (Phenylalanyl-tRNA Synthetase). Clinical features of ASyS:
  - **ILD**: ILD is the most significant clinical manifestation linked to ASyS. The presence of these antibodies, particularly anti-Jo-1, anti-PL-7, and anti-PL-12, often indicates a high risk for developing, an often rapidly progressive and life-threatning, ILD. ILD typically presents as nonspecific interstitial pneumonia (NSIP) or usual interstitial pneumonia (UIP).
  - **Myositis**: Whilst a common feature, the degree of myositis can vary widely.
  - **Other Features**: Common clinical features in patients with anti-synthetase antibodies include Raynaud's phenomenon, non-erosive arthritis, and mechanic’s hands. Systemic manifestations such as fever and weight loss are also frequently reported.

#### Myositis-associated antibodies (MAAs)

MAAs are less specific to DM and may also occur in patients with overlap syndromes involving myositis and other connective tissue diseases. These antibodies can provide additional context for disease severity and associated complications:

- **Anti-U1-RNP**: Seen with overlap syndromes, especially with mixed connective tissue disease (MCTD).
  o **Clinical Features**: Patients often present with symptoms of myositis along with features of SLE, SSc, or RA.
- **Anti-PM/Scl**: Associated with polymyositis-scleroderma overlap syndrome.
  o **Clinical Features**: Patients may have features of both myositis and scleroderma, including muscle weakness and skin thickening.
- **Anti-Ku**: Linked to overlap syndromes involving SLE and systemic sclerosis.
  o **Clinical Features**: Associated with muscle weakness, joint involvement, and features of systemic sclerosis.

Early detection of MSA and MAA autoantibodies can guide evaluation of patients, leading to initiation of immunosuppressive therapies to manage myositis and/or ILD when warranted. Additionally, monitoring for the development or progression of ILD is essential in all IIM patients, given its significant impact on prognosis. Antisynthetase antibodies play a meaningful role in the clinical evaluation and management of DM patients.

DM is a clinical diagnosis. The EULAR/ACR Classification Criteria can be used as an aid to diagnosis however, it is a classification criterion and not validated as a diagnostic criterion [12]. Antibody testing can be useful in diagnosis and management however absence of detectable autoantibodies does not preclude the diagnosis of DM or CADM.

Antibody association with specific clinical manifestations is recapped in Table 1 below.

**Table 1:** Clinical features associated with antibodies implicated in DM

Antibod	Typical Skin Findings	Photosensitive Skin Disease	Hypo-myopathic	Interstitial Lung Disease(ILD)	Calcinosis Cutis	Severe Myopathy	Malignancy Association	Other Notable Features
Anti-Mi2	Yes	No	No	No	Rare	No	None	Facial dermatosis, shawl sign, periungual erythema, high CK, responsive to treatment
Anti-TIF1-γ	Yes	Yes	Yes	No	No	No	Strongly associated	Palmar hyperkeratosis, psoriasiform plaques, GI involvement, high cancer risk in adults
Anti-MDA5	No	No	Yes	Yes (rapidly progressive)	No	No	None	Cutaneous ulceration, painful palmar papules, panniculitis, arthritis, fever
Anti-NXP2	Yes	No	No	No	Common (in children)	Yes	Increased (in adults)	Peripheral edema, severe myositis, GI bleeding, vasculopathy
Anti-SAE1/2	Yes	Yes	Yes (initially)	Possible (mild)	No	Yes (progressive)	Unknown	Severe cutaneous disease, dysphagia, fever, weight loss
Anti-Jo1 (ASyS) (Also, PL-7, PL-12, EJ, and OJ)	No	No	No	Yes	No	Yes	None	Mechanic's hands, non-erosive arthritis, fever, Raynaud's phenomenon, overlaps with DM

### Epidemiology

DM is a relatively rare condition. Similar to many other autoimmune diseases, DM occurs more frequently in women, with the incidence increasing with advancing age [4]. In 2022, a retrospective study estimated the prevalence of dermatomyositis to be 13 per 100,000, while the overall incidence of DM is estimated to be 1.1 per 100,000 person-years after adjusting for age and sex [20]. These numbers are higher than prior estimates conducted before the EULAR/ACR Classification Criteria came out, which includes skin signs that were previously overlooked under older criteria.

The overall incidence for classic DM and CADM was 0.7 and 0.5 per 100,000 person-years, respectively [20]. Higher rates have been observed in urban areas and areas with higher levels of airborne pollution [21, 22]. With an estimated 2,858 new cases diagnosed in the United States annually, DM remains a rare but potentially severe disease requiring early recognition and intervention to curtail morbidity and mortality [20].

### Etiology

As seen with most rheumatologic and autoimmune diseases, etiology includes a genetic susceptibility component and an environmental trigger (Table 2).

**Table 2:** Etiology of dermatomyositis

Etiology Category	Subcategories
Genetic Predisposition	Genetic susceptibility due to specific gene variants and polymorphisms
Environmental Factors	Proximity to the equator, UV radiation
Drugs	Aromatase inhibitors (e.g., anastrozole, letrozole, exemestane)
	Immune checkpoint inhibitors (e.g., nivolumab, pembrolizumab, ipilimumab)
	Statins (e.g., simvastatin, atorvastatin, pravastatin)
	TNFα inhibitors (e.g., etanercept, infliximab, adalimumab)
	Other drugs (e.g., hydroxyurea, penicillamine, lacosamide)
Vaccinations	Vaccines (e.g., COVID-19, influenza, hepatitis B, smallpox)
Infections	Viral infections (e.g., SARS-CoV-2, Epstein-Barr, hepatitis B/C)
	Bacterial infections
Herbal Supplements	Immunostimulatory supplements (e.g., spirulina, chlorella, green algae, echinacea)
Paraneoplastic Syndromes	Associated cancers (e.g., melanoma, lung, breast, ovarian cancers)
Occupational Exposures/Pollution	Exposure to pollutants (e.g., vehicle emissions, silica, cigarette smoke)
Miscellaneous Factors	Seasonal variation, silicone implants, liquid silicone filler rhinoplasty, hormonal changes (e.g., pregnancy)

#### Genetic predisposition

Genetic predisposition plays a significant role in susceptibility to DM. Specific genetic variants and polymorphisms have been linked to a heightened susceptibility to developing DM. Studies have identified several genetic markers that may influence disease onset and progression, suggesting a hereditary component to its etiology [4].

#### Environmental factors: proximity to equator, UV radiation

Environmental factors, particularly ultraviolet (UV) radiation, have been implicated in the pathogenesis of DM. Research indicates that individuals living closer to the equator, where UV radiation is more intense, exhibit a higher prevalence of DM and associated autoantibodies, such as anti-TIF1γ [23]. UV exposure is believed to contribute to the disease by inducing skin damage and subsequent autoimmune responses.

#### Drugs

- Aromatase inhibitors

Aromatase inhibitors, used primarily in the treatment of breast cancer, have been implicated in the development of IIMs, including DM. Specific drugs in this class, such as anastrozole, letrozole, and exemestane, have been reported to induce DM in some patients [24].

- Immune checkpoint inhibitors

Immune checkpoint inhibitors targeting the PD-1/PD-L1 and CTLA-4 pathways have been implicated in the development of dermatomyositis. Agents such as PD-L1 inhibitors (atezolizumab, avelumab, durvalumab) and PD-1 inhibitors (nivolumab, pembrolizumab, cemiplimab) have shown associations with disease onset [25, 26]. Additionally, CTLA-4 inhibitors such as ipilimumab have also been implicated [25].

- Statins/hydroxymethylglutaryl Co-A reductase inhibitors

Statins, commonly used for managing hyperlipidemia, have been reported to induce DM. The first reported case of statin-induced DM was linked to pravastatin in 1992 [27]. Other statins, including simvastatin, lovastatin, pravastatin, atorvastatin and fluvastatin, have also been associated with the disease [26].

- Tumor necrosis factor α (TNFα) inhibitors

TNFα inhibitors, used in the treatment of various autoimmune conditions, have been reported to cause DM. Drugs such as etanercept, infliximab, and adalimumab are associated with this adverse effect [26, 28]. These medications may exacerbate a DM flare or trigger new-onset DM through their immunomodulatory effects.

- Other miscellaneous drugs

Several other drugs have been implicated including hydroxyurea, penicillamine, lacosamide, phenylbutazone, cyclophosphamide, etoposide, imatinib mesylate, interferon-α2b, omeprazole, phenytoin, tegaflu, alfuzason, and gemfibrozil [26].

#### Vaccinations

Certain vaccinations have been linked to the onset of DM. This includes the COVID-19 vaccine, as well as historical vaccines such as those for influenza, hepatitis B, smallpox, tetanus, diphtheria, diphtheria with scarlet fever, diphtheria-pertussis-tetanus (DPT), tuberculosis (BCG), and inactivated polio. Studies have reported cases of DM following these vaccinations, albeit the overall risk appears to be low and therefore physicians should encourage patients to get vaccinated unless contraindicated [29, 30]. Exacerbations of DM after COVID vaccine have been reported. [31].

#### Infections

- Viral Infections

Viral infections can be potential triggers for DM. Viruses such as SARS-CoV-2, influenza, parvovirus B19, coxsackie B virus, polyomavirus, Epstein-Barr virus, hepatitis B/C, and HIV have all been implicated [4, 30].

- Bacterial Infections

There is also evidence linking bacterial infections to DM, although these associations are less well-defined compared to viral infections [4].

#### Herbal supplements

Certain herbal supplements have been reported to induce DM. Supplements such as spirulina, isalean, echinacea, chlorella, alfalfa, aphanizomenon flos aquae, elderberry, and ashwaganda are among those linked to disease onset. These supplements may act as immunostimulants, potentially triggering autoimmune responses [10, 32, 33].

#### Paraneoplastic syndromes

DM can occur as a paraneoplastic syndrome associated with various malignancies. Common cancers linked to DM include melanoma, head and neck cancers, hepatocellular carcinoma, lung cancer, lymphatic, hematopoietic, ovarian, breast, colorectal, cervical, bladder, nasopharyngeal, esophageal, pancreatic, colon, and kidney cancer, amongst others [4, 6, 8]. The presence of DM may serve as an early indicator of underlying malignancy and warrants a workup based on risk factors and age-appropriate cancer screening [6].

#### Occupational exposures/environmental pollution

DM has been associated with exposure to certain environmental pollutants and occupational hazards. Factors such as particulate matter from vehicle and industrial emissions, silica, biological and mineral dusts, gases, fumes, cigarette smoke, and nitrogen dioxide are correlated with elevated susceptibility to developing DM [21, 34].

#### Miscellaneous

Other factors potentially contributing to DM precipitation and flare include seasonal variation, liquid silicone filler rhinoplasty, silicone breast implants, and hormonal changes related to pregnancy and postpartum [4, 35].

### Pathogenesis

The pathogenesis of DM is multifaceted, involving a variety of inflammatory processes. As these mechanisms may differ between different organ system involvement, this section will primarily focus on recent developments in elucidating the pathogenesis of DM in the skin.

#### Immune populations in DM skin

Biopsies of cutaneous DM have shown hyperkeratosis, epidermal basal cell vacuolar degeneration, keratinocyte apoptosis of the basal and suprabasal layers, epidermal thinning, and dermal mucin deposition [36, 37]. Chronic, nonspecific interface dermatitis at the dermal–epidermal junction and a perivascular lymphocytic infiltrate are also commonly seen [37].

Early staining studies identified CD4 + T cells and macrophages as the primary cell populations detected in the lesional infiltrate, as well as a lack of B cells and elevated numbers of pDCs [38–41]. More recently, highly multiplexed mass cytometry has been utilized to further clarify these immune populations, with the identification of myeloid cells as the most common type, especially CD14 + macrophages, CD11 + myeloid dendritic cells (mDCs) and CD14 + CD16 + macrophages. Overall, four distinct monocyte-macrophage subsets were identified, with CD14 + macrophages showing a positive correlation with Cutaneous Dermatomyositis Disease Area and Severity Index (CDASI) scores, highlighting their potential role in driving disease activity [42]. Myeloid DCs interacted the most with other immune cells and showed elevated expressions of IL-4 and IL-13, which positively correlated with increased itch score, suggesting their clinical relevance in symptom development [42]. Increased itch in DM skin has also been shown to correlate with elevated IL-31, with CD4 + cells as the most common IL-31 producing cell type [43].

DM has classically been described as a T cell driven disease, with T cells consistently identified as a prominent cell type present in affected skin [44]. Previous studies have identified CD4 + CD40L + T cell mediated damage as the main mechanism leading to development of skin lesions, with decreased FOXP3 + Treg populations in DM skin compared to other inflammatory skin diseases [39, 45]. T cell recruitment has been linked to reduced expression of *C1orf106* and *COG8*, and elevated expression of *EVPL, GIMAP6,* and *IFI6* in the skin [44]. Mass cytometry analysis describes a dominant circulating memory T cell phenotype as well as a separate IL31 + CD4 + population which independently positively correlated with disease severity, underscoring the role of T cells in DM pathogenesis [42].

B cells contribute to DM pathogenesis, as evidenced by elevated B cell counts and increased BAFF levels in both peripheral blood and affected muscles [46–48]. Using mass cytometry, B cells have recently been shown to be enriched in DM skin compared to healthy controls, however they continue to be the least abundant immune population present [42]. Interestingly, DM-associated autoantibodies typically present with associated clinical phenotypes including skin findings, despite the relative paucity of B cells in DM skin [5, 49].

#### Cytokines

##### Interferons (IFNs)

IFNs play a prominent role in the pathophysiology of DM. Elevations in interferon stimulated genes (ISGs) were first described in muscle biopsies from both juvenile and adult DM patients, and were distinct from other inflammatory myopathies like polymyositis [50, 51]. The IFN gene response was subsequently identified in DM peripheral mononuclear blood cells (PBMCs) and skin [52, 53]. 21 out of the 25 most highly overexpressed genes in lesional DM skin were upregulated by IFN, and these genes were not overexpressed in non-lesional skin, indicating an IFN signature specific to active DM skin disease [53]. This signature was further identified to be more similar with a type I IFN induced gene response pattern and correlated with IFNβ expression in the skin and blood, rather than IFNα [53, 54]. DM cutaneous disease activity has been strongly linked to a type I IFN signature in patient blood, particularly elevated levels of IFN-β and CXCL10 in adults [55–57]. Immunohistochemistry has shown increased staining of IFNβ, as well as IFN responsive genes such as MxA in dermatomyositis skin, and this higher level of expression can differentiate DM from other inflammatory skin diseases such as eczema [58, 59]. Elevated type I IFN activity has also been associated with ILD and presence of anti-MDA-5 antibodies, whereas lower activity is typically observed in patients with anti-Mi-2 antibodies [57].

Interestingly, IFNα shows no correlation with disease activity or the IFN signature in DM [53, 55]. Analysis of two DM skin microarray datasets have identified enrichment of both type I and type II IFN pathways, correlating with prior reports of IFN signature correlation with IFNγ expression[53]. Significant elevation in IFNκ, a type I IFN isoform, has also been identified in DM lesions [42, 60].

Almost all inflammatory populations present in DM skin have shown elevated IFNβ, featuring CD4 + and CD8 + T cells, CD14 +, CD14 + CD16 +, MAC387 + and pSTING + macrophages, mDCs, and pDCs [42]. Myeloid DCs from both blood and skin produce IFNβ in DM patients, and those who have cutaneous disease refractory to hydroxychloroquine treatment have significantly more skin derived mDCs [61]. Surprisingly, while pDCs have classically been associated with type I IFN signaling and produce high levels of IFNβ in DM peripheral blood, mass cytometry identified the enriched pDC population in DM skin expressed IFNγ but not IFNβ [42, 61]. This may reflect a dysregulated or exhausted autoimmune phenotype that contributes to the type II IFN signature present in DM skin [62]. However, the mDC population is appreciably more robust in DM skin, and together with macrophages drive the IFNβ signature [42].

While the driving factors of IFN secretion in DM are incompletely understood, various causes have been identified. Viral and bacterial infections have long been reported to be a trigger for DM development [63, 64]. Skin from patients with SARS-COV-2 infection have significant transcriptome signature overlap with DM skin, with key shared genes including IFN response genes [65]. DM patients have also been shown to have upregulated retrotransposon mRNA expression compared to healthy controls, which was likely due to epigenetic factors including hypomethylation of the retrotransposon promoters and dysregulated methylation enzymes [66, 67]. While retrotransposon mRNA expression was also positively correlated with *IFNβ1* expression, ISGs were not uniformly upregulated [66, 67].

Immunostimulatory herbs, like spirulina, have recently been reported to be associated with DM onset or exacerbation, and patients with DM consume spirulina nearly 4 times more often than healthy controls or other autoimmune patients [33, 68, 69]. In vitro spirulina stimulation of PBMCs from DM patients show increased IFNβ secretion by classical monocytes and monocyte derived dendritic cells compared to healthy controls [69]. IFNγ secretion was also increased, likely secondary to activation of the TLR4-MyD88 pathway [69].

Extracellular vesicles (EVs) have also been demonstrated to be involved in autoimmune pathogenesis. Patients with IIM, including DM, have increased serum concentrations of EVs and immune cell derived extracellular vesicles [70]. Recently, EVs derived from DM patients contain a higher concentration of small EVs compared to healthy control. These small EVs correlated with the CDASI score, and induced STING mediated IFNβ secretion by PBMCs, likely due to their captured DNA content [70].

##### IL6

IL6 is a pleiotropic cytokine which plays roles in various biological and inflammatory processes, including acute phase response, infection, and autoimmunity. IL6 is elevated in DM serum compared to healthy controls, but may be less highly elevated than in other inflammatory conditions such as RA. Serum levels of IL6 correlate with disease activity as well as the type I IFN signature [71]. EVs from DM patients increased IL6 secretion by PBMCs relative to healthy control vesicles [70]. While a recent study did not show increased *IL6* mRNA expression in DM skin compared to healthy controls, *IL6* levels in lesion DM skin correlated with disease activity and itch scores [72].

##### IL17

IL17 is a major pathogenic cytokine in various autoimmune and inflammatory diseases, and elevations in the IL17 signaling pathway including phosphorylated STAT3, IL17, and the amount of Th17 cells are amplified in DM patients [73]. Interestingly, T cells isolated from DM skin secrete more IL17A than those derived from muscle [74]. Serum IL17A concentration correlates with cumulative cutaneous manifestations including facial rash, “V-neck” sign, and “shawl” sign, as well as increased inflammatory cytokines IL6 and IFNγ [75].

##### IL18

IL18 is an inflammatory cytokine which interacts with IL12 to produce IFNγ. It is elevated in DM patients and correlated with the severity of muscle inflammation, with decreases after immunosuppressive treatments [76]. IL18 is upregulated in DM skin, and keratinocytes are the major source [60]. Keratinocytes release IL18 in the context of DM triggers including UV and microbials [60, 77].

##### TNFα

TNFα plays an important function as a pathologic mediator of autoimmune diseases. It is elevated in muscle and skin biopsies of DM patients [72, 78]. TNFα correlates with increased pruritus [72]. DM patients are more likely to have TNFα promoter polymorphism which promotes an exaggerated TNFα response to UV [79]. More recently, spirulina was shown to stimulate the secretion of TNFα in the PBMCs through the TLR4 and NFkB signaling pathways, with significantly higher numbers of TNFα positive classical macrophages and macrophage-derived DCs in DM relative to healthy controls [69]. DM derived EV stimulation of PBMCs also resulted in increased TNFα secretion than those from healthy controls [70].

#### Autoantibodies

Greater than 60% of IIM patients have positive MSAs, but this number may approach 80–90% in DM patients [5]. These autoantibodies correlate with IFN levels. For example, lesional skin of anti-MDA-5 DM has significant elevations in IFNκ, as well as serum elevations of IFNα [80]. Interestingly, higher anti-MDA-5 antibody titer has been shown to significantly correlate with increased type I IFN scores, and anti-MDA-5/RNA immune-complexes induce IFNα production by TLR7 stimulation of pDCs [81]. Monoclonal antibodies derived from B cells of anti-MDA-5 DM patients directly stimulate IFNγ production; however, none of these autoantibodies recognized MDA-5 [82].

### Pathogenesis based treatments

Common mainstay treatments for DM include systemic glucocorticoids, antimalarials (hydroxychloroquine, chloroquine, and quinacrine), methotrexate, mycophenolate, and IVIG, amongst others. In addition to these therapies, more targeted treatments are becoming available. In this section we will review the standard of care therapies and emerging therapeutics.

#### Glucocorticoids

Systemic glucocorticoids are standard treatments for a variety of inflammatory disorders due to their potent immunosuppressive and anti-inflammatory activities. Downstream they suppress transcription factors responsible for regulating the production of pro-inflammatory mediators by macrophages, eosinophils, lymphocytes, mast cells, and dendritic cells [83].

Although no clinical trials have been conducted to show the effectiveness of corticosteroids in DM specifically, patients have improved with glucocorticoid treatment, with the preferred glucocorticoid being oral prednisone [4]. The steroid regimen should be tailored to the patients’ disease severity, and slowly tapered based on response. Glucocorticoids are associated with many side effects, including osteoporosis, glaucoma, hyperglycemia, insomnia, weight gain, and bruising, among others. Hence, glucocorticoid -sparing drugs are initiated concomitantly keeping the side effects of prolonged glucocorticoid therapy in mind. Patients with predominantly skin disease may not require use of glucocorticoids.

#### Antimalarials

Hydroxychloroquine (HCQ) is a first line treatment for amyopathic DM patients [84]. If HCQ doesn’t work, then quinacrine can be added or HCQ changed to chloroquine. Their exact mechanism of action remains unclear, they exhibit a range of immunomodulatory, anti-inflammatory, and antiproliferative properties [85, 86]. Due to their basic nature, antimalarials can cross cell membranes and enter acidic cytoplasmic vesicles [85–87]. One proposed mechanism for suppressing cytokine production involved in the inflammatory response is the elevation of lysosomal pH. As the pH increases, the binding of antigenic peptides with MHC class II molecules is blocked, reducing presentation to CD4 + T lymphocytes [85–87].

##### Hydroxychloroquine (HCQ)

An initial study on patients with recalcitrant cutaneous DM found that all patients responded well to the addition of HCQ, and three patients had total resolution of their skin lesions. However, no effects on myositis were observed [88]. A retrospective study found beneficial effects of antimalarials on cutaneous manifestations of DM, with nine of 17 patients responding positively and seven experiencing near full recovery [89]. The most common side effects of HCQ are gastrointestinal upset, difficulty sleeping and concentrating [84]. Approximately 11.5% of DM patients have a skin reaction to HCQ that can manifest as urticarial, eczematous, or exacerbation of DM lesions [90]. However, a more severe side effect is retinal toxicity, which warrants routine ophthalmologic visual field examination at baseline and after five years, and then on a yearly basis after that, along with more sensitive testing techniques [90, 91].

##### Chloroquine (CQ)

HCQ is a derivative of CQ, sharing the same core mechanisms of action albeit HCQ is generally safer and better tolerated. HCQ is primarily used as the agent of choice given its relatively less toxic side effect profile especially when it comes to retinal toxicity. CQ requires more frequent eye monitoring than HCQ, with routine practice of every 4–6 months [90].

##### Quinacrine (QC)

When given along with HCQ or CQ, QC has a synergistic effect and demonstrated therapeutic promise for recalcitrant disease. In a study, QC was beneficial for 50% of DM patients not responding to or tolerating HCQ [92, 93]. Compared to HCQ and CQ, QC is linked to reversible yellow discoloration of the skin [92, 93]. It lacks the risk of ocular toxicity, eliminating the need for routine visual field monitoring if used without HCQ or CQ [90].

#### Methotrexate (MTX)

Used for both classic and amyopathic DM, MTX is an effective steroid-sparing immunosuppressant. First used in rheumatology to treat RA, MTX works by inhibiting an enzyme, causing obstruction of adenosine metabolism. The accumulation of adenosine causes down-stream anti-inflammatory effects: downregulation of B-cells, inhibition of T-cell activation, and blockade of interleukin binding to its cell surface receptor [94].

Studies have revealed both cutaneous symptoms and myositis improved after receiving MTX [95]. In one study, patients with steroid‐resistant inflammatory myositis were administered oral MTX. Of these 25 patients, 88% showed marked clinical improvement and 43% successfully tapered their glucocorticoid dose [96]. Further investigation demonstrated that low dose MTX can reduce or clear the cutaneous manifestations of DM [97]. Data on the risk of adverse events is variable, and with side effects ranging from nausea, hepatotoxicity, and teratogenicity, factors such as liver/renal function and family planning should be assessed for patients considering MTX [4, 95]. Its effectiveness has made MTX a first line treatment for patients’ intolerant of antimalarial therapy or with severe cutaneous and muscle disease.

#### Azathioprine (AZA)

AZA is a steroid sparing therapy commonly used in chronic inflammatory diseases. It is a purine analog that blocks purine synthesis, which inhibits cellular replication and causes immunosuppressive effects. Several studies have noted AZA’s efficacy in inflammatory myositis [98, 99]. In a controlled double-blind study, patients receiving both glucocorticoids and azathioprine (AZA) demonstrated superior long-term outcomes over three years than glucocorticoids alone [99]. A more recent study observed improved 10-year survival in patients who began treatment with MTX versus those treated with AZA; however, this difference was not upheld in multivariable analysis across the full duration of follow-up [100]. In a different cohort, use of AZA was negatively associated with mortality [101]. Patients on AZA need to be monitored for bone marrow suppression, hepatic enzyme elevations, and renal impairment. Side effects include GI intolerance and flu-like symptoms [99].

#### Mycophenolate mofetil (MMF) and mycophenolic acid

MMF is another systemic immunosuppressant that functions by inhibiting inosine monophosphate dehydrogenase, a key enzyme in the de novo synthesis of purines [102]. It induces apoptosis of activated T-lymphocytes and can downregulate the expression of adhesion molecules, reducing lymphocyte and monocyte infiltration into areas of inflammation [102]. It has become one the preferred treatment options due to its lymphocyte specificity enabling a decreased toxicity profile [103].

In patients who have pulmonary involvement, MMF is a first-line treatment [104]. A retrospective study found that 3 of the 4 patients who had ILD with DM had complete normalization of pulmonary function at 1-year follow-up [105]. Case reports and uncontrolled studies have shown the effectiveness of MMF for both skin disease and myositis [105, 106]. Common adverse effects include GI issues, increased risk of infection, and increased risk of malignancy [107]. Routine blood testing is needed to monitor specifically for leukopenia and transaminitis.

#### Calcineurin inhibitors

##### Tacrolimus

Tacrolimus is a calcineurin inhibitor that selectively inhibits the activation of T lymphocytes. This drug has shown promise in DM patients with ILD and those resistant to conventional therapies. A recent meta-analysis of 9 studies comprising a total of 491 patients who were given oral tacrolimus in combination with steroids showed improvement in pulmonary function (specifically forced vital capacity) and overall survival [108]. Additionally, no significant increase in infections or renal dysfunction was reported when compared to standard therapy. Unlike broader immunosuppressants, tacrolimus may offer a more targeted immunomodulatory approach with a favorable safety profile. Despite promising results reported in the meta-analysis, the heterogeneity of study designs and limited randomized controlled trials underscore the need for further high-quality research into the use of tacrolimus for DM. Topical formulations have shown efficacy in treating the skin manifestations of DM as well [109].

##### Pimecrolimus

Pimecrolimus is a topical calcineurin inhibitor which has been used for the dermatological manifestations of DM as well [110]. Both topical pimecrolimus and tacrolimus may have similar therapeutic benefits compared to mid-strength corticosteroids without the associated risks of skin thinning or pigmentary changes [109]. Therefore, these topical alternatives may be a favorable choice for lesions in sensitive areas like the face.

#### Intravenous immunoglobulin (IVIG)

IVIG consists of pooled immunoglobulins and is commonly used to treat autoimmune diseases [111]. It is believed that the main mechanism of IVIG is related to the blockade of the FcγR receptors for IgG, thereby resulting in the clearance of autoantibodies [112]. It may additionally act by limiting complement consumption and interfering with assembly of the membrane attack complex (MAC) [112, 113]. In DM, C3 activation leads to formation and deposition of MAC on capillaries, leading to their destruction and subsequent microangiopathy [113, 114]. There is terminal complement within the vessels of the skin among patients with DM, demonstrating an important mechanism that research is currently targeting [115]. Additionally, IVIG can alter gene expression in the muscles of DM patients, leading to downregulation of cytokines and chemokines [113, 116].

Previous studies demonstrated improving muscle strength with IVIG [4]. Recently, IVIG has also been shown to improve the cutaneous manifestation of treatment-resistant DM [117]. Headache, fluid overload, and infusion-related reactions are the most frequently reported side effects of IVIG [117, 118]. A more severe adverse event is thromboembolism; in one study, six patients in a group of 47 patients receiving IVIG experienced a thromboembolic event [118]. Slower rates of infusion decrease the possibility of clotting. With IVIG's therapeutic potential for both muscle and cutaneous disease, the Food and Drug Administration (FDA) and European Medicines Agency have approved IVIG (Octagam 10%) administration for DM [117].

### Janus Kinase (JAK) inhibitors

The aforementioned treatments represent nonspecific anti-inflammatory therapy, creating a need for targeted therapies. One such option includes JAK inhibitors, which block the Type 1 IFN pathway, resulting in the downstream activation of the JAK/STAT cascade [119]. In vivo studies show that activation of the IFN pathway upregulated genes was linked to atrophy, impaired the formation of capillaries, and reduced the number of endothelial cell junctions. However, pre-treatment with JAK inhibitors prevented type I IFN–mediated damage to both muscle and endothelial cells [119].

There are currently five FDA-approved JAK/STAT inhibitors, two of which have been used off label to treat DM: tofacitinib and ruxolitinib. The first reported JAK inhibitor used therapeutically to treat DM was ruxolitinib, during which the patient regained muscle strength and her skin lesions resolved [120]. An open label trial of refractory, mostly skin-predominant DM patients treated with tofacitinib also showed marked improvement in both cutaneous and muscle disease [121]. Following these findings, tofacitinib has been increasingly utilized and studied, revealing significant improvement of DM in some patients. Tofacitinib has been tested in patients with anti-MDA5-positive CADM, demonstrating a significantly improved survival rate compared to controls receiving alternative immunosuppressive treatments [122]. However, while some studies found minimal side effects, others report an increased risk of viral infections [122]. There is an ongoing phase 3 trial of a TYK2 inhibitor for IIM [123].

### Rituximab (RTX)

RTX is a monoclonal antibody targeting the CD20 antigen expressed on pre-B and mature B lymphocytes. It promotes lymphocyte depletion through four main mechanisms: antibody-dependent cellular cytotoxicity, complement-dependent cytotoxicity (CDC), antibody-mediated phagocytosis and direct induction of apoptosis [124]. It is effective in treating muscle involvement in DM including refractory myositis and additionally has been shown to improve DM-associated interstitial lung disease [125].

A small uncontrolled pilot trial with patients receiving RTX found that patients increased in muscle strength and other signs such as rash and alopecia also improved [125]. Another open label trial did not show improvement in the skin in DM [126]. The Rituximab in Myositis randomized trial did not find a difference in skin response between those who received placebo and those who received RTX [127]. As such, RTX is mostly used to treat muscle symptoms. In these trials, infections were a common side effect, while infusion reactions rarely occurred [125, 127]. Patients positive MSAs have generally demonstrated strong therapeutic responses, indicating greater effectiveness of B cell–directed treatments in this subgroup [127]. Individuals with anti–Mi-2 or anti–Jo1 exhibited more pronounced clinical improvement compared to those with other MSAs [127].

### Interferon pathway targets

#### Type 1 IFN

The role of IFN has been discussed previously in the pathogenesis section of this article. As a result, anti-IFNα antibodies have been formulated to target this inflammatory pathway. A clinical trial assessing sifalimumab, a monoclonal antibody targeting IFNα, in DM patients showed a reduction in IFN gene signature levels in both blood and muscle tissue [128]. Another recent case report described improvement of DM with the use of the IFNAR1-blocking antibody anifrolumab [129]. A phase 2 trial of a monoclonal antibody directed against human IFNβ was found to improve the skin and decrease muscle enzyme levels in patients with refractory muscle-predominant DM [130].

#### Anti-IFNβ (Dazukibart)

Dazukibart demonstrated significant reductions in disease activity and was well tolerated overall, highlighting IFNβ inhibition as a compelling therapeutic strategy for adults with DM [130].

#### Toll-like Receptors (TLR)

In DM, TLRs are expressed in inflammatory myopathic tissues. Specifically, TLR7 and TLR8 is upregulated in infiltrating leukocytes, indicating their essential role in initiating and sustaining a systemic autoimmune response targeting skeletal muscle [131]. Because TLRs are important in inducing the production of type I IFNs, they are targets to reduce disease activity. Enpatoran, a dual TLR7/8 inhibitor under development for several autoimmune diseases and is currently undergoing evaluation in a phase II clinical trial for DM [132].

#### Lenabasum

Lenabasum is an oral cannabinoid receptor Type 2 agonist. Expressed on activated immune cells, fibroblasts, and endothelial cells, this G protein–coupled cannabinoid receptor plays a role in dampening inflammation and fibrosis when activated, as demonstrated in multiple inflammatory disease animal models [133]. A recent clinical trial in amyopathic DM found that after receiving Lenabasum, DM patients had greater improvement in CDASI activity and multiple efficacy outcomes [133]. A phase 3 trial of Lenabasum in mostly classic DM patients failed to demonstrate significant improvement using an outcome measure that mainly captures muscle disease. The results in the patients with skin-predominant DM were similar to the successful phase 2 trial [134, 135].

#### Chimeric antigen receptor (CAR) T cell

CAR-T cells are novel therapeutic options for autoimmune diseases. By depleting autoreactive B-cells, CAR T cells have been shown to be an effective treatment for SLE [136]. A patient with myositis and ASyS refractory to treatment had significant improvement with CD19-targeting CAR T cell treatment [137]. With a clinical trial currently in progress, CAR T cells provide a potential future treatment.

#### Efgartigimod

By binding to the neonatal Fc receptor, efgartigimod inhibits its interactions with IgG, causing a reduction in IgG recycling and degradation of IgG and pathological autoantibodies, without altering other immunoglobulins levels [138]. In a humanized mouse model, efgartigimod reduced circulating IgG levels, suggesting potential therapeutic effects for myositis patients [138]. A therapeutic trial of this mechanism is ongoing.

#### Complement inhibitors

Complement (C5b-9) mediated endothelial injury, driven by terminal-MAC deposition leads to capillary loss in muscle tissue, fibrosis, necrosis, and muscle fiber atrophy [139]. Eculizumab, a monoclonal antibody targeting C5 that inhibitsboth C5a generation and membrane attack complex formation, showed some efficacy in treating refractory DM patients [140]. Ravulizumab is another C5 complement inhibitor approved for treatment for various complement-mediated disorders, with a global phase II/III randomized, placebo-controlled trial in progress to evaluate the efficacy and safety of Ravulizumab [141].

#### Perspectives

Future perspectives for DM evaluation and treatment are increasingly focusing on precision medicine, enhanced outcome measures, and targeted therapies. The Treatment Improvement Score (TIS), currently used as the primary outcome for DM trials, is largely a measure of muscle disease. Its use has led to exclusion of the large number of amyopathic or post-myopathic DM patients from most trials. The development of more specific and sensitive outcome measures like the Dermatomyositis Outcomes for Muscle and Skin (DMOMS), aims to better capture patient experiences, disease activity, and therapeutic response [16]. This shift reflects the need for tools that can comprehensively assess cutaneous and muscle components separately, alongside patient-reported outcomes. This will allow inclusion of both classic DM and CADM patients in trials, so that the full spectrum of the disease can be studied. Future research will likely emphasize validating these metrics across diverse populations to improve standardization in clinical trials [10, 16].

Not all patients require a muscle biopsy for diagnosis. Clinical symptoms with a compatible MRI and/or EMG can be sufficient or further supported with the help of a less invasive skin biopsy in those with cutaneous manifestations [142].

On the treatment front, promising advancements are being made with targeted therapies, including JAK inhibitors and monoclonal antibodies against key pathways like Type I IFN. These agents have demonstrated efficacy in reducing muscle enzyme levels, improving muscle strength, and controlling skin manifestations, particularly in refractory DM cases​. As more targeted drugs are tested, the hope is to minimize reliance on broad-spectrum immunosuppressants, which often have significant side effects.

In terms of research, the integration of biomarkers and genomic profiling will play a crucial role in identifying patient subsets that may respond better to certain therapies [10]. Additionally, the increasing recognition of environmental triggers and their interaction with genetic predispositions in DM may lead to preventative strategies in at-risk populations. Overall, the future of DM research is geared toward personalizing therapy, improving outcome measures to aid in medication trials, and discovering new therapeutic targets that address both immune dysregulation and tissue repair.

## Data Availability

Data sharing not applicable to this article as no datasets were generated or analyzed for this study.
